# The Role of Animal Models in Developing Pharmacotherapy for Obstructive Sleep Apnea

**DOI:** 10.3390/jcm8122049

**Published:** 2019-11-22

**Authors:** Lenise Jihe Kim, Carla Freire, Thomaz Fleury Curado, Jonathan C. Jun, Vsevolod Y. Polotsky

**Affiliations:** Division of Pulmonary and Critical Care Medicine, Department of Medicine, Johns Hopkins University School of Medicine, Baltimore, MD 21224, USA; lkim38@jhmi.edu (L.J.K.); cfreire2@jhmi.edu (C.F.); tfleury2@jhmi.edu (T.F.C.); jjun2@jhmi.edu (J.C.J.)

**Keywords:** obstructive sleep apnea, pharmacotherapy, animal models, upper airway anatomy, neuromuscular response, loop gain, arousal threshold

## Abstract

Obstructive sleep apnea (OSA) is a highly prevalent disease characterized by recurrent closure of the upper airway during sleep. It has a complex pathophysiology involving four main phenotypes. An abnormal upper airway anatomy is the key factor that predisposes to sleep-related collapse of the pharynx, but it may not be sufficient for OSA development. Non-anatomical traits, including (1) a compromised neuromuscular response of the upper airway to obstruction, (2) an unstable respiratory control (high loop gain), and (3) a low arousal threshold, predict the development of OSA in association with anatomical abnormalities. Current therapies for OSA, such as continuous positive airway pressure (CPAP) and oral appliances, have poor adherence or variable efficacy among patients. The search for novel therapeutic approaches for OSA, including pharmacological agents, has been pursued over the past years. New insights into OSA pharmacotherapy have been provided by preclinical studies, which highlight the importance of appropriate use of animal models of OSA, their applicability, and limitations. In the present review, we discuss potential pharmacological targets for OSA discovered using animal models.

## 1. Introduction

Obstructive sleep apnea (OSA) is characterized by cyclical obstruction of the upper airway causing intermittent cessation or reduction of airflow during sleep [1]. Epidemiological studies have shown that moderate to severe OSA affects up to 33% to 50% of the adult population [2,3]. Continuous positive airway pressure (CPAP) is the first-line therapy for OSA [4]. However, OSA patients frequently do not tolerate CPAP therapy, showing a poor adherence to treatment [5]. Approximately 50% of OSA patients do not use CPAP during sleep or use it insufficiently (less than 4 h/night) [5,6]. Second-line treatment approaches, including oral appliances and surgery of the upper airway, can be alternatives for CPAP, but their efficacy is variable. Low compliance with CPAP and the lack of efficacious alternatives highlight the importance of pharmacotherapy development for OSA. Although a great effort towards a pharmacological approach has been made over the recent years, there is still no approved medication for OSA.

**Figure 1:**
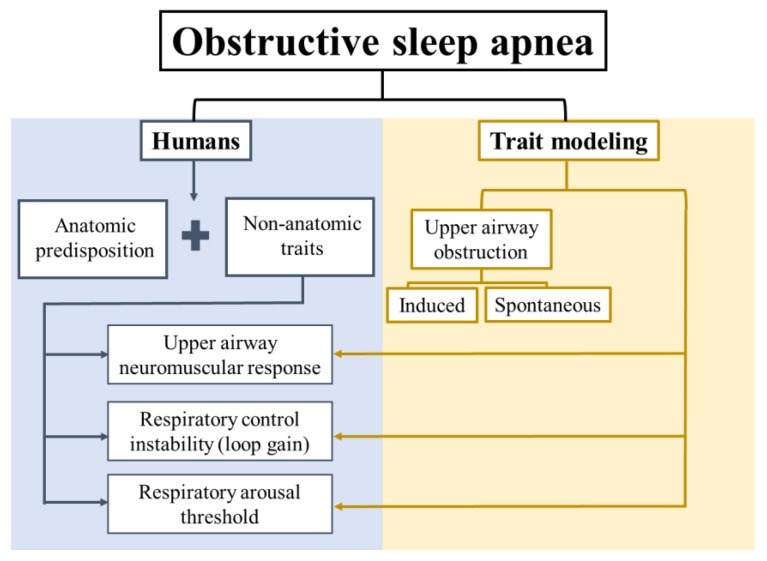
Schematic representation of the anatomical and non-anatomical traits of obstructive sleep apnea (OSA) pathophysiology in humans, and a general classification of OSA traits modeling.

At least four major traits contribute to the development of OSA [7,8,9,10,11]: (1) Impaired upper airway anatomy, (2) impaired upper airway neuromuscular response, (3) respiratory control instability (loop gain), and (4) low respiratory arousal threshold (Figure 1). Anatomic predisposition is necessary but not sufficient for OSA development [9], since obstructive events do not occur during wakefulness. This highlights the role of non-anatomical traits, and the potential to target these traits with pharmacotherapy. Animal models have been useful for an understanding of OSA pathogenesis and identification of potential pharmacological targets. Investigators have focused either on the development of animal models of upper airway obstruction during sleep, or modeling one of the predisposing traits (upper airway neuromuscular responses, respiratory control, or sleep continuity—Figure 1). In this review, we will discuss current insights into potential pharmacotherapy of OSA based on animal models.

## 2. Upper Airway Anatomy or Collapsibility

### 2.1. Definition and Human Evidence

Anatomic predisposition is one of the key factors for the development of pharyngeal collapse and consequent OSA [7,8,9,10,11]. Anatomic factors include craniofacial abnormalities and obesity. Several studies have shown that OSA patients have craniofacial morphology that predisposes to a narrow, crowded, and collapsible upper airway. Reduced pharyngeal airway space, inferiorly placed hyoid bone, and increased anterior facial height distinguished adults with OSA from controls [12]. Volumetric magnetic resonance imaging (MRI) showed that patients with OSA have enlarged lateral pharyngeal walls, tongue, and total soft tissues surrounding the upper airway compared to control patients [13]. Consequently, the average airway area and lateral and anterior–posterior dimensions were significantly smaller in OSA patients [13]. Shortened cranial base and altered size and position of the maxilla and mandible are also associated with narrowing of the pharyngeal area and a higher risk of OSA [12,14,15]. 

Measurement of upper airway dimensions using imaging techniques is often performed during wakefulness and does not necessarily imply the functional impairment of the upper airway during sleep [11]. Rather, the luminal pressure at which the upper airway collapses (Pcrit) is the gold standard metric to assess of the propensity of the upper airway to close during sleep [9,16,17,18]. A negative Pcrit implies that the airway remains open, even when negative nasal pressure is applied; while a positive Pcrit implies that the airway opens only when positive airway pressure is applied. In patients with normal upper airway anatomy, Pcrit values below −5 cmH_2_O are necessary to protect from pharyngeal airway collapse and OSA [11,19]. Higher Pcrit values reflect a more collapsible upper airway and OSA patients with severe anatomical compromise generally show Pcrit values > 0–2 cmH_2_O [9,19]. Pcrit values differ depending on body position and sleep state, with increased collapsibility in the supine position and in rapid eye movement (REM) sleep [20,21]. Pcrit can be further divided into two more specific parameters. First, the passive Pcrit quantifies collapsibility when airway reflexes are not engaged. Passive Pcrit is solely predicated upon static upper airway anatomy. Second, the active Pcrit quantifies collapsibility when airway reflexes are engaged by flow limitation, leading to increased motor tone. The Johns Hopkins and Harvard laboratories developed a set of research techniques to measure passive and active Pcrit [22]. 

Obesity contributes to pharyngeal collapse during sleep and is a well-known risk factor for OSA [20]. The adipose tissue deposition in structures surrounding the upper airway may reduce the pharyngeal airspace. Obese patients with OSA showed more fat deposition in the soft palate compared to weight-matched controls without OSA [23]. Patients with OSA have larger pharyngeal fat pad areas compared to control subjects [13,24,25]. Increased tongue volumes and greater fat deposition at the base of the tongue can also be observed in obese patients with OSA compared to obese controls [26]. Central adiposity can also contribute to the anatomical predisposition of the upper airway to OSA. The abdominal accumulation of adipose tissue reduces lung volumes and promotes caudal traction of the pharynx, increasing the collapsibility of the upper airway [7,27,28,29]. Conversely, weight loss diminishes upper airway collapsibility. A body mass index (BMI) reduction by 17% decreased the Pcrit from 3.1 ± 4.2 to −2.4 ± 4.4 cmH_2_O in OSA patients [30].

Although many OSA therapies attempt to reverse the dysfunction of upper airway, the anatomy of the upper airway is generally not affected by pharmacologic manipulation [31], with the exception of weight loss [32] as well as diuretics in heart failure patients, which can reduce rostro-caudal fluid shifts [33]. Nevertheless, animal models with different patterns of pharyngeal anatomic impairment have been used to test potential pharmacotherapies for OSA.

### 2.2. Animal Models Focused on Upper Airway Anatomic Predisposition to OSA

Spontaneous OSA has been identified in several animals [34]. The English bulldog has a narrow upper airway with a large soft palate and was one of the first animals exhibiting a breathing pattern similar to that of human OSA [35]. OSA in bulldogs was present predominantly in REM sleep. However, it was not associated with obesity. By contrast, Yucatan minipigs develop obesity-related OSA [36]. In addition, several animal models used mechanical upper airway obstruction [37,38,39,40,41,42]. An interesting feline model of OSA observed in the supine position with the neck flexed mirrors positional apnea in humans [38]. However, the utility of these models for pharmacotherapy is uncertain.

Obese rodents exhibit features of sleep apnea, including impaired upper airway anatomy or function, and changes in breathing patterns during sleep. Obese Zucker rats (body weight ~600 g) have reduced pharyngeal cross-sectional area during both expiration and inspiration [43], and a greater amount of fat infiltration in the tongue muscle compared to non-obese Zucker rats [44]. New Zealand obese mice (NZO), which exhibit leptin resistance and metabolic syndrome, have increased visceral fat compared to lean mice. NZO mice have fat deposits in the pharyngeal soft tissues, tongue, soft palate, and upper airway walls [45]. Recently, Baum and colleagues [46] showed that NZO mice had a larger number of spontaneous apneas and hypopneas than lean New Zealand black mice. However, the study did not distinguish between obstructive and central events since sleep studies were conducted without the assessment of respiratory effort. 

Our group developed a system to measure Pcrit in anesthetized mice by inducing ramp decreases in nasal pressure while measuring expiratory airflow obstruction, obviating the need for monitoring tracheal pressure [47]. Obese mice showed a higher passive Pcrit than lean mice [48]. Our group also developed a plethysmography system initially described by Hernandez and colleagues [49], which has the capacity of recording sleep and breathing in freely behaving mice. We have shown that both leptin-deficient *ob*/*ob* mice, diet-induced obesity mice (DIO), and NZO mice exhibit sleep disordered breathing, characterized by marked increases in inspiratory flow limitation (IFL) during REM sleep [49,50,51]. *ob*/*ob* and DIO mice also exhibited hypoventilation, with a higher partial pressure of carbon dioxide (PaCO_2_) during wakefulness than lean mice [51,52]. Taken together with the imaging findings described above, we can conclude that obese rodents have compromised upper airway anatomy and increased collapsibility, leading to OSA. However, these rodent models may not be ideal models of OSA due to their mild phenotype (primarily flow-limited breathing and obstructive hypopneas, without complete obstructive apneas), which is predominantly expressed in REM sleep [51,53]. Obese rodent models have yet to be leveraged for the development of pharmacotherapy. 

## 3. Upper Airway Neuromuscular Response

### 3.1. Definition and Human Evidence

OSA develops when there is inadequate neuromuscular response to pharyngeal obstruction during sleep. One of the major determinants of upper airway patency is tongue muscle tone. The tongue is composed of extrinsic and intrinsic muscle groups, both of which are innervated by the hypoglossal nerve [54]. Extrinsic muscles are largely responsible for the tongue movements, whereas the intrinsic muscles maintain its shape. The extrinsic tongue muscles fall in two main categories: Protrudors that move the tongue forward, including the genioglossus (GG), a major pharyngeal dilator; and retractors that move the tongue backwards towards the pharyngeal wall [54]. Hypoglossal motor neuron activity initiates and maintains contraction, while its absence causes relaxation, of nearly all the muscle fibers in the tongue. Pivotal work by Remmers and colleagues [55] clarified the role of neuromuscular input to the GG in the pathophysiology of human OSA. The lack of GG motor input favored tongue prolapse and oropharynx occlusion while motor activation of the GG counteracted pharyngeal closure.

Lingual muscles work in synergy with the GG to maintain airway patency. In rodents, hypercapnic stimulation increased retractor (styloglossus and hypoglossus) muscle tone, suggesting that both muscle groups play a role in stabilizing tongue structures when ventilatory drive is high [56]. In humans, investigators documented different activation patterns between wakefulness and sleep [57]. At sleep onset, tongue protrudor and retractor activity decreases [31]. In response to airway obstruction, GG muscle activity increases above wakefulness level, and retractor activity decreases [57]. Studies in rats showed that tongue protrudor and retractor co-activation is superior to protrudor activation alone to maintain pharyngeal patency [56,58]. The role of GG tone on upper airway patency is also illustrated by hypoglossal nerve stimulation therapy, which uses an electrical impulse generator to recruit upper airway regional muscles and treat OSA [59].

As in humans, the rodent tongue functions as a muscular hydrostat controlled by axonic projections of motor neurons arranged in a similar fashion in the hypoglossal nucleus. These similarities enable us to utilize rodent models to understand the role of neuromuscular responses in the pathophysiology of OSA, and to develop pharmacotherapies. Authors have focused on two main outcomes to characterize upper airway neuromuscular responses: (1) Electromyography (EMG) and (2) airflow.

### 3.2. Animal Models of Neuromuscular Response: EMG Outcomes

Several seminal studies have examined central control of upper airway tone in animals, with GG EMG as the primary outcome. The first evidence of hypoglossal motor neuron modulation was described in an anesthetized feline with pontine carbachol injections, a cholinergic receptor agonist, to mimic REM sleep atonia [60]. Reduction in hypoglossal motoneuronal activity was mediated by reduced serotonergic excitation due to the decreased activity of medullary raphe neurons [60]. Serotonin (5-hydroxytryptamine (5-HT)) exerted excitatory effects on hypoglossal motoneurons in anesthetized rats, whereas the withdrawal of serotonergic input caused the loss of neuromuscular input under anesthesia [61]. However, Sood and colleagues [62] later demonstrated that inhibition of the serotonin axis failed to modulate genioglossus responses during natural non-rapid eye movement (NREM) and REM sleep. 

Richard Horner’s laboratory examined the effects of noradrenergic and cholinergic drugs on GG activation during natural NREM and REM sleep. In rats, Chan and colleagues [63] examined the effects of noradrenergic hypoglossal antagonism or stimulation mediated by terazosin and phenylephrine, respectively (Table 1). Perfusion of the α 1 receptor antagonist terazosin into the hypoglossal nucleus decreased GG activity while the α 1 receptor agonist phenylephrine increased GG activity during wakefulness and NREM sleep but not during REM sleep. Subsequently, Grace and colleagues [64,65] showed that GG muscle tone in REM sleep was maintained by muscarinic receptors, and muscarinic inhibition was functionally linked to inwardly rectifying potassium channel (GIRK) activation. The blockade of GIRK channels in the hypoglossal motor pool with barium augmented tonic and respiratory-related GG activity in rats, reversing the upper airway hypotonia during NREM and REM sleep [66] (Table 1). 

Song and Poon [67] provided another line of evidence that noradrenergic stimulation plays an important role in GG muscle control. The investigators used yohimbine, an antagonist of presynaptic α2-adrenerereceptor (i.e., a noradrenergic agonist). Yohimbine significantly increased GG muscle tone in anesthetized rats (Table 1). Taken together with the data from Horner’s lab in sleeping rats, the study provides credible evidence that noradrenergic drugs may be useful in OSA. These experiments laid the foundation for a recent human clinical trial utilizing a combination of the noradrenergic agonist, atomoxitine, and a muscarinic blocker, oxybutinine [77]. Nevertheless, both classes of drugs have significant systemic adverse effects on cardiovascular, gastrointestinal, and urogenital systems, limiting their clinical use. 

In more recent experiments, activation of the hypoglossal motor pool was accomplished by introduction of bioengineered receptors, called designer receptors exclusively activated by designer drugs (DREADDs) [78]. DREADDs are G-protein-coupled human cholinergic receptors that have been genetically modified to be recognized only by specifically engineered ligands [79]. Fleury Curado and colleagues stereotactically injected adeno-associated viruses carrying DREADDS into the hypoglossal motor pool [68]. Eight weeks after virus administration, activation of these DREADDS induced significant pharynx dilation throughout the respiratory cycle (Table 1). These results re-emphasize the principle that selective modulation of the hypoglossal motor pool may maintain upper airway stability, but the use of gene therapy as treatment for OSA remains uncertain.

The reliance on EMG responses as the primary outcome may be problematic. First, GG EMG activation is not tantamount to airway patency; airflow measurements are required to determine whether GG recruitment could improve OSA. Second, other muscles innervated by the hypoglossal nerve (tongue retractors and intrinsic muscles) as well as other cranial nerves (glossopharyngeal and vagus) are likely to be involved and have not been sufficiently studied. Successful drug development for OSA will require attention to both of these limitations. 

### 3.3. Animal Models of Neuromuscular Response: Airflow Outcomes

Translational models have been used to demonstrate the impact of anatomical or neuromuscular interventions on airflow. In rabbits, Lee and colleagues [40,42] paralyzed the GG muscle with botulinum toxin type A and evaluated polysomnography and upper airway dynamics using computed tomography (CT) scans during drug-induced sleep. Two weeks after injection, the number of apneas and hypopneas increased and the diameter of the upper airway at the level of the palate and tongue base was diminished. In anesthetized animals, electrical stimulation of the hypoglossal nerve increased airflow and upper airway patency [58,80,81,82,83,84,85,86], laying the foundation for the development of electrical-stimulating devices for OSA. In 1993, Schwartz and colleagues [81] utilized an isolated feline upper airway model to modulate GG activity. Hypoglossal nerve stimulation reduced Pcrit and increased flow. These translational models have advanced our understanding of the pathogenesis of OSA but have yet to be leveraged for pharmacotherapy. 

A major limitation of translational OSA research is the need for anesthesia, which is likely to affect upper airway muscle tone and breathing. The Hopkins group developed a system for continuously recording polysomnographic signals in unanesthetized, unrestrained mice [49]. They modified a whole-body plethysmography chamber with features, including an open-circuit design and an air bladder upon which the mouse rests, to enable extended monitoring and assessment of tidal volume, airflow, and respiratory effort. Polotsky and colleagues used this system to measure Pcrit and associated GG EMG activity, airflow, and effort in a variety of mouse models, including leptin-deficient *ob*/*ob* [50,70], DIO [51], and NZO mice [49]. DIO and NZO mice exhibited IFL during REM sleep [49,50,51] and higher passive Pcrit values than lean mice [48]. A more severe OSA phenotype was proposed by Fleury Curado and colleagues [87] by silencing hypoglossal motoneurons in lean C57BL/6J mice using inhibitory DREADDS. The DREADDS were delivered bilaterally to the hypoglossal nucleus, and inhibitory activity was then induced by intraperitoneal injection of the DREADD ligand, clozapine-N-oxide (CNO). These mice developed flow-limited breaths during both REM and NREM sleep, which would correspond to snoring or obstructive hypoventilation in children [87,88,89].

Another rodent model of OSA is the leptin-deficient *ob*/*ob* mouse. Leptin is secreted by adipose tissue into the circulation. It crosses the blood–brain barrier (BBB) and acts in hypothalamic centers to increase metabolic rate and suppress food intake [90]. *ob*/*ob* mice and leptin receptor-deficient *db*/*db* mice are hyperphagic, hypometabolic, and severely obese [91]. It was previously shown that *ob*/*ob* mice have hypercapnia and reduced CO_2_ sensitivity, which resolves with leptin replacement [52,92] (Table 1). More recently, leptin has been shown to have a role in controlling upper airway patency [50,69,70]. *ob*/*ob* mice exhibit inspiratory flow limitation, especially during REM sleep. Subcutaneous leptin improved upper airway patency and increased ventilatory drive, with no significant change in passive Pcrit [50,69] (Table 1). To localize the site of leptin’s activity, Yao and colleagues [70] injected leptin into different brain regions. They took advantage of the unidirectional rostral–caudal flow of cerebrospinal fluid by administering leptin in the lateral as opposed to the fourth ventricle. Only leptin injections in the lateral ventricle relieved upper airway obstruction while both routes of leptin administration stimulated ventilation (Table 1). This suggests that leptin acts in the dorsomedial hypothalamus to relieve upper airway obstruction, and the hindbrain (nucleus of the solitary tract) to upregulate ventilation. 

In subsequent work, our group have shown that DIO mice develop sleep disordered breathing, despite high circulating leptin levels and leptin resistance [51]. Moreover, these findings were consistent with reports in obese patients [93,94]. Leptin resistance has multiple mechanisms, but limited permeability of the BBB plays a key role [95,96,97,98]. Berger and colleagues [71] administered leptin intranasally in mice in an effort to bypass the BBB. Intranasal leptin increased ventilation during NREM and REM sleep and decreased the number of oxygen desaturation events in REM sleep (Table 1). By contrast, intraperitoneal leptin did not significantly improve breathing during sleep. Thus, central leptin signaling may be a future target for OSA drug development. 

## 4. Respiratory Control Instability (Loop Gain) 

### 4.1. Definition and Human Evidence

During wakefulness, breathing is regulated by both conscious respiratory drive from supra-pontine brain structures, and autonomic chemoreflex control [99]. During sleep, respiratory drive is mainly governed by chemoreflexes, with the fluctuations in CO_2_ levels representing the major stimulus to breathe [11,99]. The magnitude of the ventilatory response to CO_2_ during sleep can drive oscillations in breathing, leading to arousals during respiratory disturbances [11]. “Loop gain” (an engineering concept) describes the amplitude of ventilation in response to a disturbance, as might occur with obstruction of the upper airway [99]. There are two major components that regulate loop gain: (1) A controller gain and (2) plant gain [100]. Controller gain predominantly refers to chemoreflex sensitivity to arterial blood gases while the plant gain reflects the capacity of the respiratory system to alter gas exchange. A delay also exists between the detection of blood gas fluctuations by chemoreceptors (controller gain) and ventilatory responses to these fluctuations (plant gain), which can initiate and propagate ventilatory stability [100]. 

Different techniques have been developed to assess loop gain in OSA patients. Wellman and colleagues [8,10] developed a non-invasive method to measure OSA traits, including the gain of the ventilatory control system, by intermittently dropping CPAP from optimum pressures, in which the upper airway is completely opened, to several subtherapeutic pressures for 3 to 5 min during sleep. At suboptimum CPAP pressures, there is compensatory ventilation, in which the respiratory drive increases but does not reach eupneic levels, because of the upper airway obstruction. In this method, loop gain is quantified as the compensatory ventilatory response (ΔVentilation) divided by the increase in ventilatory drive (ΔVentilatory drive) [8,10]. However, all of the currently available techniques only quantify the general loop gain of OSA patients and cannot provide a clear estimation of controller and plant gains.

In patients with anatomical predisposition to upper airway collapse, high loop gain causes instability, thereby increasing the severity of OSA [9,101]. A high loop gain was observed in 36% of patients with high anatomic predisposition to OSA defined by a Pcrit between −2 and +2 cmH_2_O [9]. High loop gain might lead to an over-exuberant respiratory response to obstruction, triggering large negative inspiratory pressure swings and collapse of the pharyngeal upper airway. In addition, hyperventilation may diminish the activity of hypoglossal motoneurons, leading to upper airway collapse [9,10,11,102,103]. However, it is still unclear if (1) OSA patients have a higher loop gain compared to non-OSA individuals and (2) whether a high loop gain is inherent to OSA or is induced by OSA traits. As extensively reviewed by Deacon-Diaz and Malhotra [99], the findings comparing loop gain between OSA and non-OSA patients are still contradictory. However, evidence from studies controlling for key confounding factors (i.e., obesity and CPAP status) have suggested a higher loop gain in apneic individuals [104]. 

### 4.2. Animal Models of Ventilatory Instability in OSA

Mice do not inherently have OSA but may develop sleep disordered breathing when their ventilatory control system is perturbed. Intermittent hypoxia caused by apneic events augments the ventilatory response to hypoxia (HVR) [105,106,107]. Exposure to intermittent hypoxia can induce neuroplasticity of motoneurons involved in respiratory control, including the phrenic and hypoglossal neurons [108]. The sustained increase in ventilatory neural activity induced by intermittent hypoxia that persists after hypoxic stimulation has been termed long-term facilitation (LTF). LTF reduces CO_2_ levels below eupnea, leading to an increase in the controller gain [99,109,110,111]. Prabhakar’s laboratory has examined LTF and its contribution the development of an OSA phenotype in rodents. Rats exposed to nine episodes of hypoxia (5% O_2_) for 10 days (8 h/day) had a 48% increase in HVR, analyzed by efferent phrenic nerve activity [106]. 

The carotid body is a critical organ of oxygen sensing and altering its function can lead to disordered breathing patterns. Prabhakar’s laboratory found that LTF was related to increased HVR (i.e., chemosensitivity) in carotid bodies [112]. Transcriptional activator hypoxia-inducible factor 1 alpha (HIF-1α) has been proposed as a key molecule in the regulation of carotid body plasticity and HVR. In wild-type mice, preconditioning to hypoxia for 3 days increased the HVR [113]. However, Hif1α^+/−^ mice showed a reduced HVR and the absence of carotid body activity in response to hypoxia, suggesting the role of HIF-1α in the ventilatory adaptation and carotid body plasticity to chronic hypoxia. Peng and colleagues [72] investigated the effects of the gasotransmitters, carbon monoxide (CO) and hydrogen sulfide (H_2_S), on carotid body activity, HVR, and induction of sleep apneas. They showed that the deficiency of CO augmented apneas and hypopneas during NREM and REM sleep in mice. On the other hand, lack of H_2_S by genetic ablation or pharmacological blockade of the H_2_S-synthesizing enzyme normalized breathing and prevented central and obstructive apneas in mice and rats (Table 1). These findings demonstrate that altered ventilatory control can lead to sleep apnea, and suggest that one approach to treating OSA may be blocking H_2_S release.

Recently, our lab discovered that the carotid bodies respond to leptin, with downstream effects on minute ventilation and HVR [73]. We showed that the long functional isoform of leptin receptor (LepR^b^) is expressed in approximately 74% of glomus cells in carotid body and that leptin infusion increased carotid sinus nerve activity in vivo. In lean C57BL/6J mice, subcutaneous infusion of leptin increased minute ventilation and HVR, and these leptin effects were abolished after carotid body denervation. In obese LepR^b^-deficient *db*/*db* mice, transfection of LepR^b^ in the carotid body also increased minute ventilation and HVR (Table 1). Similar findings of leptin-modulating HVR were also observed in obese Zucker rats [74] (Table 1). Overall, our results indicate that leptin is a potent ventilatory stimulant, increasing HVR by acting on the LepR^b^ expressed in carotid bodies. Our data suggest that the modulation of leptin signaling could be a target for pharmacotherapy. However, excessive carotid body activity by leptin administration may in theory destabilize respiratory control, causing apneas. Leptin also has several peripheral effects, such as increasing blood pressure [96,114], which may limit its use for OSA.

## 5. Respiratory Arousal Threshold 

### 5.1. Definition and Human Evidence

Respiratory arousals contribute to the pathophysiology of OSA, destabilizing both the control of breathing and upper airway function [7,11]. Thus, preventing respiratory arousals (i.e., increasing the arousal threshold) might be a pharmacological target for OSA. Studies in humans using sedative-hypnotic drugs to achieve this goal have yielded inconsistent results [115,116,117,118,119,120,121]. Eckert and colleagues [119] showed that Eszopliclone was effective in reducing the apnea-hypopnea index in individuals with a low arousal threshold (stage 2 arousal threshold between 0 and −15 cmH_2_O) but not in those with a higher arousal threshold (stage 2 arousal threshold = −40 ± 6; overall range −25 to −63 cmH_2_O). An arousal threshold in recent studies was determined based on the respiratory effort inducing arousal [122]. Thus, pharmacological agents that increase the arousal threshold may be used in patients with a low arousal threshold. 

### 5.2. Animal Models Focusing on Arousal Threshold

Stimuli that provoke arousal in OSA include CO_2_ elevation and airway occlusion. Studies in rodents, dogs, and piglets utilized progressive hypercapnia during sleep to quantify the arousal threshold as a function of CO_2_ levels. Park and colleagues demonstrated that sedative hypnotics increased the arousal threshold and increased GG muscle activity when administered systemically, but decreased GG activity when the same drugs were administered locally to the hypoglossal nucleus [75,123] (Table 1). 

Saper and his group [124,125,126] described the neural circuitry responsible for respiratory arousals in mammals. Interestingly, all the chemosensory and mechanical stimuli converge upon the external lateral parabrachial nucleus (PBel) in the pons. By utilizing selective optogenetic activation and inhibition they were able to modulate hypercapnia-induced arousals and identify sites in the forebrain that receive input from the PBel [76] (Table 1). Thus, the PBel and its network are promising cellular targets for interventions to promote respiratory stability during sleep.

## 6. Conclusions and Future Perspectives

OSA is a complex disorder caused by factors, including altered upper airway anatomy, upper airway neuromuscular responses, respiratory control, and respiratory arousal threshold. Animal models have advanced our understanding of how these factors contribute to the pathogenesis of OSA. These models have revealed potentially druggable targets for OSA, including (1) the role of leptin in the control of breathing, (2) the role of noradrenergic agonists and antimuscarinic agents in GG muscle control, (3) the role of serotoninergic input to the hypoglossal nucleus during sleep, and (4) the role of sedative hypnotics. Modern techniques, such as DREADDs and optogenetics, are promising tools to investigate the pathophysiology of OSA and may yield novel targets for OSA pharmacotherapy.

## Figures and Tables

**Table 1:** Summary of findings about potential pharmacotherapy for obstructive sleep apnea from studies using animal models.

Phenotypic Features	Study	Animal Model	Pharmacotherapy Findings
Neuromuscular responses of upper airway	Chan et al. [63]	Wistar rats	- Microdialysis perfusion of terazosin, an α_1_ receptor antagonist, into the hypoglossal nucleus decreased GG activity - Infusion of α_1_ receptor agonist phenylephrine augmented the GG activity during wakefulness and NREM sleep but not in REM sleep
	Grace et al. [64,65,66]	Wistar rats	- Perfusion of scopolamine hydrobromide, a muscarinic-type acetylcholine receptor antagonist, into hypoglossal nucleus generated GG muscle suppression during REM sleep - Muscarinic inhibition was linked to GIRK activation - Blockade of GIRK channels in hypoglossal motor pool increased tonic activity of GG during sleep
	Song and Poon [67]	Sprague-Dawley rats	- Yohimbine, a presynaptic α_2_-adrenergic antagonist, reversed the decrease in hypoglossal motoneuron activity
	Fleury Curado et al. [68]	Lean C57BL/6J mice	- DREADDs activation with CNO in hypoglossal nucleus increased GG activity and markedly dilated the pharynx
	Polotsky et al. [69]	Leptin deficient *ob*/*ob* mice	- *ob*/*ob* mice treated with leptin showed a decrease in the frequency of inspiratory flow limitation and an increase in maximum inspiratory airflow with no changes in Pcrit
	Pho et al. [50]	Leptin deficient *ob*/*ob* mice	- Subcutaneous infusion of leptin increased minute ventilation and maximum inspiratory airflow
	Yao et al. [70]	Leptin deficient *ob*/*ob* mice	- Intracerebroventricular leptin administration into lateral ventricle attenuated the inspiratory flow limitation and obstructive hypopneas in *ob*/*ob* mice - Leptin effects on ventilation were localized in dorsomedial hypothalamus and the nucleus of the solitary tract
	Berger et al. [71]	Diet-induced obesity (DIO) mice	- Intranasal administration of leptin augmented the ventilation during NREM and REM sleep, and reduced the number of oxygen desaturations in REM sleep
Respiratory instability (high loop gain)	Peng et al. [72]	HO-2-null mice and SHR rats	- HO-2-null mice have increased apneas and hypopneas during REM and NREM sleep - Genetic ablation of CSE normalized breathing in HO-2-null mice - Pharmacological blockade of CSE with L-propargyl glycine treated apneas in HO-2-null mice and SHR rats
	Caballero-Eraso et al. [73]	Lean C57BL/6J and LepR^b^ deficient *db*/*db* mice	- Leptin infusion increased minute ventilation and HVR in C57BL/6J mice - Carotid body denervation abolished the leptin effects on ventilation - Transfection of LepR^b^ in the carotid body of *db*/*db* mice increased minute ventilation and HVR
	Yuan et al. [74]	Zucker rats	- Leptin injection for 7 days increased minute ventilation and HVR - The effects of leptin on ventilation were abolished after carotid body denervation
Respiratory arousal threshold	Park et al. [75]	Wistar rats	- Systemic administration of the sedative-hypnotics lorazepam and zolpidem increased the GG activity during sleep - Lorazepam and zolpidem increased the arousal threshold and the GG activity immediately before an arousal - Central administration of lorazepam and zolpidem into the hypoglossal motor nucleus suppressed the GG activity
	Kaur et al. [76]	CGRP-CreER mice	- Optogenetic inhibition of PBel^CGRP^ neurons prevented arousal to CO_2_

GG: genioglossus muscle; NREM: non-rapid eye movement; REM: rapid eye movement; GIRK: G protein-coupled inwardly rectifying potassium channels; DREADDs: Designer Receptor Exclusively Activated by Designer Drugs; CNO: Clozapine-N-oxide; Pcrit: passive critical closing pressure; HO-2: heme oxygenase 2; CSE: cystathionine-γ-lyase; SHR: spontaneously hypertensive rats; LepR^b^: long functional isoform of leptin receptor; HVR: hypoxic ventilatory response; CGRP: calcitonin gene-related peptide; PBel^CGRP^: external lateral parabrachial nucleus; CO_2_: carbon dioxide.
